# Firefighters, posttraumatic stress disorder, and barriers to treatment: Results from a nationwide total population survey

**DOI:** 10.1371/journal.pone.0190630

**Published:** 2018-01-05

**Authors:** Jieun E. Kim, Stephen R. Dager, Hyeonseok S. Jeong, Jiyoung Ma, Shinwon Park, Jungyoon Kim, Yera Choi, Suji L. Lee, Ilhyang Kang, Eunji Ha, Han Byul Cho, Sunho Lee, Eui-Jung Kim, Sujung Yoon, In Kyoon Lyoo

**Affiliations:** 1 Ewha Brain Institute, Ewha Womans University, Seoul, South Korea; 2 Department of Brain and Cognitive Sciences, Ewha Womans University, Seoul, South Korea; 3 Department of Radiology, University of Washington, Seattle, Washington, United States of America; 4 Department of Bioengineering, University of Washington, Seattle, Washington, United States of America; 5 Department of Radiology, Incheon St. Mary's Hospital, College of Medicine, The Catholic University of Korea, Seoul, South Korea; 6 Department of Psychiatry, University of Utah, Salt Lake City, Utah, United States of America; 7 Department of Psychiatry, Ewha Womans University, Seoul, South Korea; Stellenbosch University, SOUTH AFRICA

## Abstract

Repeated exposure to traumatic experiences may put professional firefighters at increased risk of developing posttraumatic stress disorder (PTSD). To date, however, the rate of PTSD symptoms, unmet need for mental health treatment, and barriers to treatment have only been investigated in subsamples rather than the total population of firefighters. We conducted a nationwide, total population-based survey of all currently employed South Korean firefighters (n = 39,562). The overall response rate was 93.8% (n = 37,093), with 68.0% (n = 26,887) complete responses for all variables. The rate of current probable PTSD was estimated as 5.4%. Among those with current probable PTSD (n = 1,995), only a small proportion (9.7%) had received mental health treatment during the past month. For those who had not received treatment, perceived barriers of accessibility to treatment (29.3%) and concerns about potential stigma (33.8%) were reasons for not receiving treatment. Although those with higher PTSD symptom severity and functional impairment were more likely to seek treatment, greater symptom severity and functional impairment were most strongly associated with increased concerns about potential stigma. This nationwide study points to the need for new approaches to promote access to mental health treatment in professional firefighters.

## Introduction

Professional firefighters respond to a variety of life-threatening perils [1] in their efforts to provide essential community services. Therefore, they are frequently exposed to traumatic events and have an increased risk for developing posttraumatic stress disorder (PTSD) [2, 3]. Previously, there have been studies that examined PTSD prevalence in firefighters who had been exposed to traumatic incidents [1, 4–7] and other studies that examined it in a subpopulation of firefighters without reference to an index trauma[3, 8]. However, as far as we know, no study has assessed the nationwide and total population of professional firefighters in regards to probable PTSD rates and status quo of receiving mental treatment. Therefore, large-scale and total population-based studies are required in this high-risk population.

Over the last few decades, there has been rapid and substantial progress in developing evidence-based treatments of PTSD that employ a variety of pharmacological, psychotherapeutic, and behavioral interventions [9, 10]. These interventions provide clinicians with diverse and efficacious treatment approaches for this potentially debilitating disorder. Treatment intervention has been shown to be a critical predictor of the illness course [11]. Despite the importance and availability of effective treatments, their actual delivery to affected individuals suffering from symptoms of PTSD may be suboptimal. Unmet treatment needs and contributing factors have been reported for war veterans [12, 13], community-based general population [14, 15], and first responders who were involved in the aftermath of the World Trade Center terrorist attacks [5]. Symptom severity has been reported as a major factor that influences individuals to seek mental health treatment [5, 14, 16, 17]. However, even in the most severely affected individuals, mental health treatment needs may be unmet [5, 17].

Stigma and perceived obstacles such as lack of knowledge about PTSD or treatment routes, and financial or time burden have been reported to play important roles as barriers to mental health treatment [18, 19]. Stigma can have detrimental influences on both an individual's reputation and career [20], and therefore prevent people from seeking mental health treatment [13]. In particular, it is a bigger concern especially in male-dominated occupations including war veterans and first responders than in other populations [21]. One of the primary concerns of veterans who seek PTSD treatment is the possibility of being held responsible for PTSD, due to their personal weakness [22]. In addition, they believe that acknowledging mental illness can be a potential threat to their careers [22]. With regard to perceived obstacles, first responders most frequently reported difficulties in scheduling an appointment, probably due to their heavy workload and shiftwork schedule [19]. However, most previous studies on barriers to mental health treatment in firefighters and other first responders have been conducted in Western countries [19].

South Korea shows lower utilization rate of mental health services in patients with mental disorders compared to Western countries [23, 24] in spite of the relatively lower financial burden due to the universal health coverage system that covers most of the standard mental health treatment costs [25]. A recent national survey reported that 22.2% of South Koreans with current or past history of mental diseases have used mental health services in their lifetime [23], compared to the one-year rate of 43.1% in the US [26]. This phenomenon may be explained by a high level of stigma that is prevalent in the Asian culture [23, 24]. Many Asians consider mental illness as a sign of weakness rather than as a biological disease that needs professional treatment [27]. Therefore, rates of access to mental health treatment among firefighters with PTSD may also be lower in South Korea than in Western countries. In addition, concerns about stigma may be a more influential factor than perceived obstacles among those with more severe PTSD symptoms or functional impairment.

In the present study, the proportion of individuals with PTSD symptoms was systematically assessed in a population-based survey of South Korean public firefighters. We further examined whether affected individuals were receiving mental health treatment and elucidated barriers that might hinder access to treatment. Specifically, we investigated whether PTSD symptom severity and perceived functional impairment are associated with mental health service use among firefighters with current probable PTSD. Although our primary analysis was conducted for treatment from any professional, sensitivity analysis was also performed for treatment from a psychiatrist since most South Korean patients with mental disorder prefer to receive treatment from a psychiatrist [23]. In addition, associations of PTSD symptom severity and perceived functional impairment with barriers to treatment such as concerns about stigma and perceived obstacles were evaluated in firefighters with current probable PTSD but who had not received treatment.

## Materials and methods

### Participants

The study population consisted of all currently employed firefighters listed in the South Korean national registry (n = 39,562) [28] who were public employees providing front-line emergency services including firefighting, performing rescues, providing emergency medical services, and carrying out desk duties at fire departments based on a job-rotation system. The study ensured strict anonymity and secure data management. The Institutional Review Board of Ewha Womans University approved the study protocol and granted a waiver of informed consent.

### Procedures

Survey response forms were sent out to all regional fire departments and distributed to participants by a contact person within each fire department, to maximize the response rate. Firefighters participated in the survey on a voluntary basis and all data were collected anonymously. Contact information was provided for participants who wanted to request a follow-up for personal assessment or referral. The survey was conducted from March to April of 2014.

### Data input

Completed forms were collected via mail and scanned electronically using Brother ADS-2600W scanners and ControlCenter4 scanning software (Bridgewater, NJ). Remark Office OMR, version 8.4 (Gravic, Inc., Malvern, PA), an optical mark recognition software, was used to automatically score multiple-choice items. If any recognition error occurred with an item or a page, the error-inducing section was reprocessed for software recognition. To detect and correct for possible errors, all data coded as blank or multiple responses were compared with the original response forms and corrected, if necessary, by an investigator blinded to the aim of the study. In case of continuous optical software recognition failure, responses were entered manually.

### Measures

In addition to demographic and career information, functional impairment in family relations was assessed with a 4-point Likert-scale question. The PTSD Checklist (PCL) [29] for the Diagnostic and Statistical Manual of Mental Disorders-IV (DSM-IV) [30], which had been validated with the Clinician-Administered PTSD Scale (CAPS) [31] for DSM-IV [30] in South Korean firefighters [32], was used to estimate the 30-day prevalence of probable PTSD. Participants were asked to complete the PCL for the most severe traumatic event they had experienced on duty. The total score of the PCL ranges from 17 to 85, with higher scores indicating more severe symptoms. Probable PTSD cases were defined by a PCL total score of 45 or greater [32]. In case of missing values, a total score of 45 or greater from non-missing questions was identified as a probable PTSD case, whereas a total score less than 45, which would not exceed 45 even if all of the missing items were scored with the maximum possible values, was recorded as a non-case. When this inference was not possible, cases with missing values were recorded as missing. We also estimated the 30-day prevalence of probable PTSD using DSM-IV algorithm-derived method for PCL as sensitivity analysis (see Supplementary Method A in S1 File). The question on perceived functional impairment due to PTSD symptoms was adopted from the Patient Health Questionnaire-9 [33].

*"Too Good"* (4 question items) and *"Suspect Questionnaire"* (2 question items) subscales from the Personality Diagnostic Questionnaire-IV were used to assess response validity [34, 35]. The *Too Good* subscale is designed to evaluate underreporting or fake-good responding, while the *Suspect Questionnaire* subscale assesses lying and responding randomly. These items were interspersed with other items throughout the questionnaire.

A questionnaire adapted and modified from Hoge and colleagues [13] was used to assess mental health treatment use during the past year and past month, and barriers to treatment. Questions regarding potential barriers to treatment were answered by those who had not received treatment. The domain of perceived obstacles to treatment was composed of four statements: "I didn't know that I can receive help for these matters," "I don't know where to get help," "I don't have enough time to spend on treatment," and "Mental health treatment costs too much money." Any positive response to these statements was regarded as having perceived obstacles to treatment. The domain of concerns about potential stigma associated with seeking treatment was composed of two statements: "It would harm my career," and "I would be seen as weak." Any positive response to these two statements was regarded as having concerns about potential stigma.

All survey instruments used in this study are presented in the Appendix A of S1 File.

### Statistical analysis

**Fig 1:**
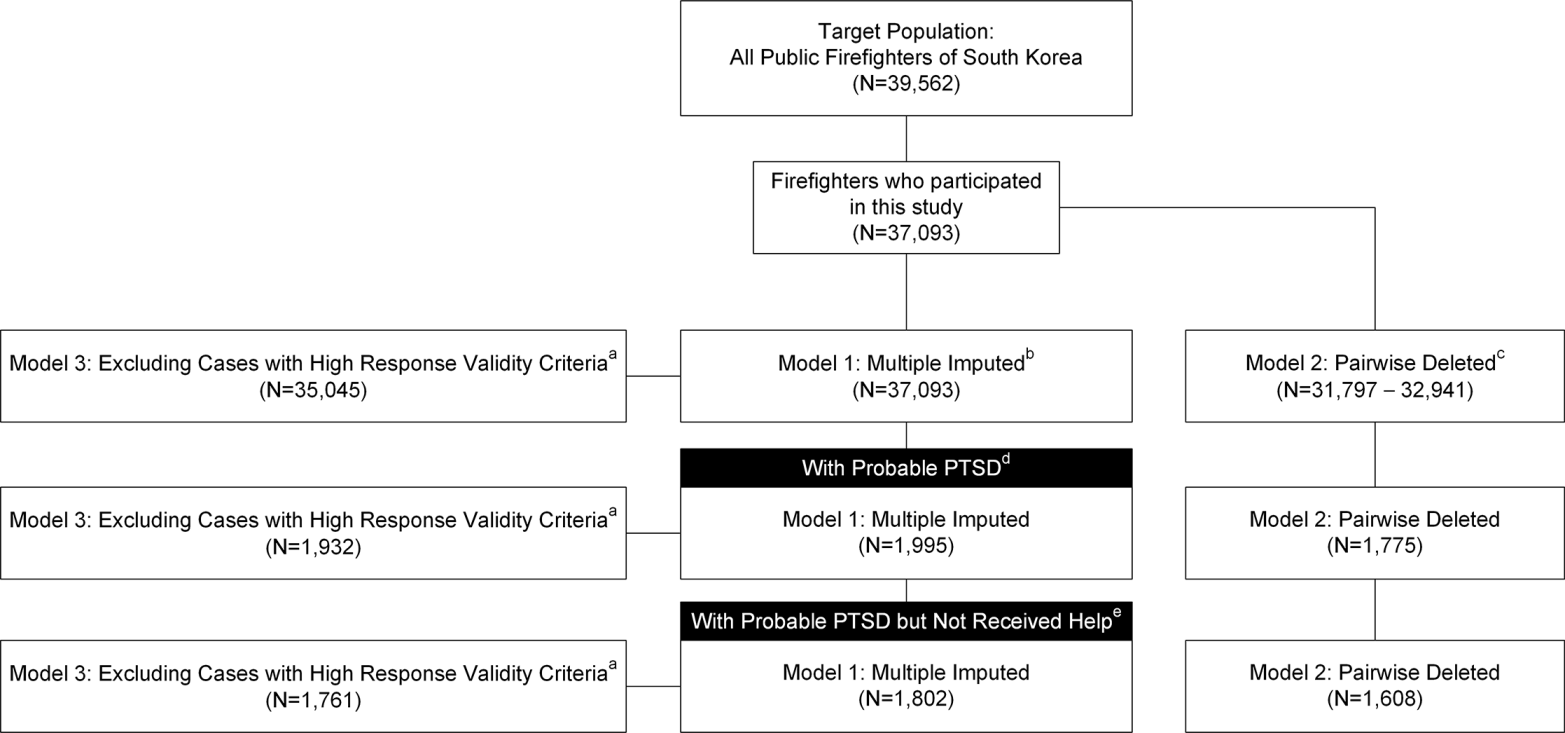
Participant response rate throughout recruitment and data analyses. ^a^ Excluded individuals who could be faking good, answering randomly, or lying. ^b^ Imputed datasets were generated using the chained equations approach [37]. ^c^ Numbers are expressed in range due to the varying numbers of missing values for each variable. ^d^ Current probable posttraumatic stress disorder (PTSD) was defined as ≥ 45 on the PTSD checklist. ^e^ Those who had not received treatment from any professional during the past month.

To handle missing data, analyses were conducted in the imputed dataset (Model 1) as well as in the original one (Model 2), since the cumulative effects of missing values in several variables may cause a loss of statistical power and precision due to excluding a substantial proportion of the respondents [36]. A multivariate imputation method that generated five imputed samples was used, through a chained equations approach [37]. In addition, the analyses were repeated in another dataset (Model 3) that excluded individuals whose responses were questionable in both *Too Good* and *Suspect Questionnaire* scales [34] from Model 1. The primary analytic statistics were from multiple imputed datasets (Model 1). The response rate and analytic strategies are summarized in Fig 1.

Descriptive statistics were derived from the imputed datasets as well as from the original dataset. Independent t-tests and Pearson chi-square tests were used to examine whether there were differences in demographic, career-related, and clinical characteristics including mental health treatment use between firefighters with and without current probable PTSD.

To examine whether symptom severity and perceived functional impairment are associated with mental health service use among firefighters with current probable PTSD [5, 14, 16, 17], logistic regression analysis was performed. The dependent variable was mental health treatment use during the past month from any professional such as a psychiatrist, general medical doctor, religious mentor or counselor. Sensitivity analyses were performed using the mental health treatment use from a psychiatrist as the dependent variable.

We examined associations of PTSD symptom severity and perceived functional impairment with barriers to treatment such as concerns about potential stigma and perceived obstacles in firefighters with current probable PTSD but who had not received treatment from any professional. Since the two variables of treatment barriers would be correlated, maximum-likelihood two-equations probit modeling [38] was performed.

For these logistic and probit models, covariates were demographic and career-related characteristics that showed significant differences between PTSD and non-PTSD groups (Table 1), as these characteristics themselves could be associated with PTSD symptom severity or perceived functional impairment. Multicollinearity among independent variables was assessed with the variance inflation factor.

**Table 1:** Characteristics of firefighters with or without current probable posttraumatic stress disorder^a^.

			PTSD group (*n* = 1,995)	Non-PTSD group (*n* = 35,098)	Test statistics^b^
Characteristic					
Demographic					
	Age–yrs.		42.6 ± 8.0	41.3 ± 8.5	*p*<0.001, d = 0.15, CI = 0.10–0.19
	Male sex–no. (%)		1,874 (93.9)	33,144 (94.4)	*p* = 0.33, OR = 0.91, CI = 0.75–1.10
Career-related–no. (%)					
	Higher rank^c^		1,273 (63.8)	20,291 (57.8)	*p*<0.001, OR = 1.29, CI = 1.17–1.41
	Working at cities^d^		1,238 (62.0)	21,882 (62.3)	*p* = 0.79, OR = 0.98, CI = 0.90–1.08
	Years since employment–yrs.		15.9 ± 8.3	14.7 ± 8.7	*p*<0.001, d = 0.15, CI = 0.10–0.19
Clinical					
	Total PCL score		54.9 ± 8.9	21.1 ± 6.9	*p*<0.001, d = 4.81, CI = 4.75–4.87
	Received treatment during the past year–no. (%)				
		From any professional	315 (15.8)	920 (2.6)	*p*<0.001, OR = 6.96, CI = 6.06–7.99
		From a psychiatrist	226 (11.3)	589 (1.7)	*p*<0.001, OR = 7.48, CI = 6.31–8.87
	Received treatment during the past month–no. (%)				
		From any professional	193 (9.7)	388 (1.1)	*p*<0.001, OR = 9.59, CI = 7.99–11.50
		From a psychiatrist	155 (7.8)	274 (0.8)	*p*<0.001, OR = 10.71, CI = 8.64–13.26
	Functional impairment in family relations				*p*<0.001, OR = 9.23, CI = 8.40–10.13
		Not at all	698 (35.5)	28,371 (81.7)	
		Slightly	772 (39.1)	5,412 (15.6)	
		Moderately	364 (18.4)	672 (1.9)	
		Extremely	141 (7.1)	282 (0.8)	

*Note*. OR = odds ratio; CI = 95% confidence interval; PCL = Posttraumatic Stress Disorder (PTSD) Checklist.

^a^ Current probable PTSD is diagnosed using the PCL (total score ≥ 45).

^b^ Independent t-tests or chi-square tests were used. Effect size was estimated using Cohen's d or odds ratio.

^c^ Fire captain or higher.

^d^ Locations with a population greater than 380,000 were defined as cities [40, 41].

Stata version 13.1 (StataCorp., College Station, TX) was used for all computations. An α level of .05 (2-tailed) was used throughout.

## Results

Among 39,562 currently employed public firefighters, 37,093 (93.8% for any response and 68.0% for complete responses) [39] replied to the survey questionnaire (Fig 1). A total of 16,699 (44.9%) firefighters were currently engaged in fire suppression; 3,615 (9.7%) in rescue work; 7,787 (20.92%) in emergency medical service; and 9,127 (24.5%) were assigned to administration. Each job was separately counted if participants reported having more than 2 jobs. Table 1 and Table A in S1 File provide information on demographic, career-related, and clinical characteristics of firefighters with and without current probable PTSD. Although group differences in age, higher rank, years since employment were significant among demographic and career-related variables, their effect sizes were too small to be considered meaningful. However, all clinical outcomes were significant with large effect sizes. Firefighters with current probable PTSD showed higher levels of total PCL score, mental health treatment use, and functional impairment in family relations than those without. We found that 5.4% (n = 1,995; 95% confidence interval [CI], 5.1–5.6) of the firefighters was identified as having probable PTSD in the past month. Sensitivity analysis found similar PTSD prevalence when the DSM-IV algorithm-derived method was applied (Supplementary Results A in S1 File).

**Fig 2:**
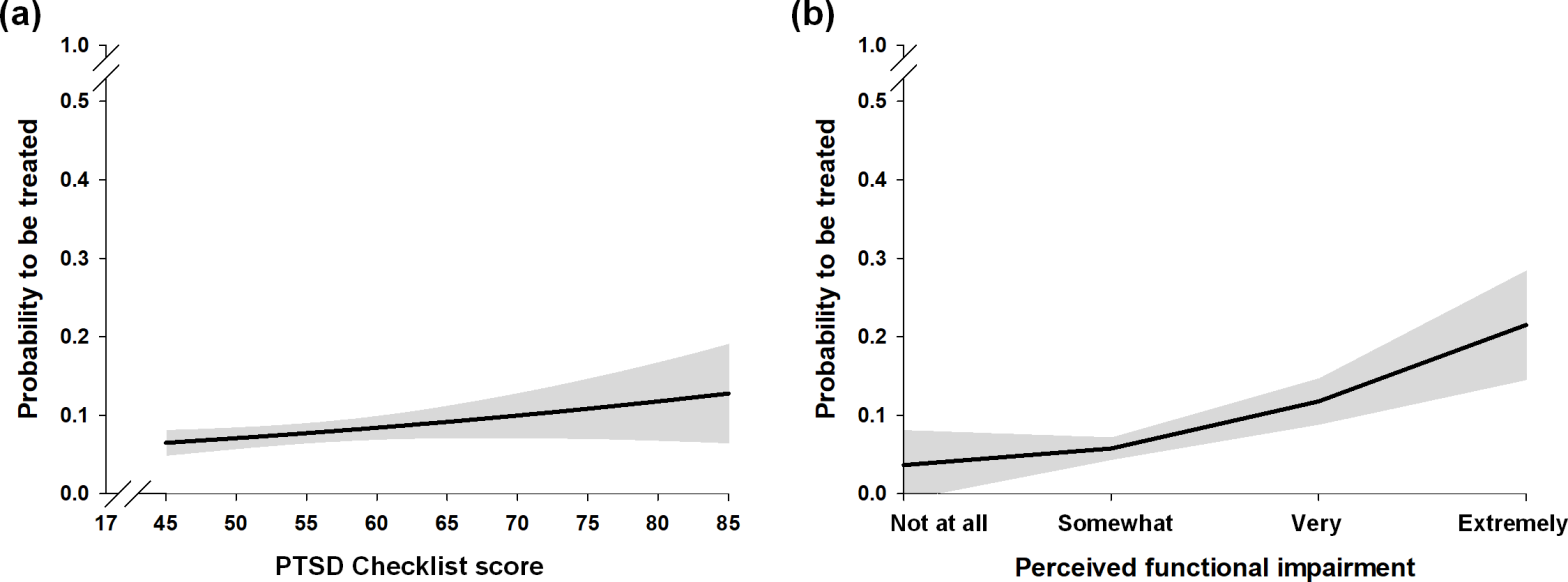
**Mental health treatment use during the past month from any professional, among firefighters with current probable posttraumatic stress disorder (n = 1,802), according to the symptom severity (a) and perceived functional impairment (b).** *Note*. PTSD = posttraumatic stress disorder.

Among all firefighters, 3.3% (95% CI, 3.1–3.5) and 1.6% (95% CI, 1.4–1.7) had received treatment for a mental health problem from any professional during the past year and past month, respectively. Among those with probable PTSD, a greater proportion had received mental health treatment from any professional: 15.8% (95% CI, 14.2–17.4) and 9.7% (95% CI, 8.4–11.0) during the past year and past month, respectively. Smaller subgroups received treatment from a psychiatrist (Table 1). Within the subgroup of firefighters with current probable PTSD, both symptom severity and perceived functional impairment were positively associated with mental health treatment use during the past month (Table 2, Fig 2, and Fig A in S1 File). Nevertheless, the majority of individuals with current probable PTSD had not received any treatment (90.3% during the past month; 95% CI, 89.0–91.6) (Fig 2).

**Table 2:** Associations of posttraumatic stress disorder (PTSD) symptom severity and perceived functional impairment with mental health treatment use in firefighters with current probable PTSD (n = 1,995).

Variable			Treatment during the past month^a^		
			Adjusted OR (95% CI)		*p* value^b^
PTSD symptom severity					
	Total PCL score, per 1-SD increase		1.18	(1.00–1.39)	0.048
	Total PCL score, per 10-point increase		1.20	(1.00–1.49)	0.048
Perceived functional impairment					
	Not at all		1.00	(referent)	-
	Somewhat		1.65	(0.45–6.01)	0.44
	Very		3.60	(1.00–13.00)	0.05
	Extremely		7.39	(1.95–28.02)	0.004

*Note*. PCL = Posttraumatic Stress Disorder (PTSD) Checklist; OR = odds ratio; SD = standard deviation; CI = confidence interval.

^a^ Treatment from any professional. Results from sensitivity analyses using treatment from a psychiatrist as outcome variables are presented in Fig A (c, f) in S1 File.

^b^ Age and rank were included as covariates, whereas years since employment was not included due to its collinearity with age (variance inflation factor = 8.7).

For those with current probable PTSD who had not received treatment during the past month, 29.3% (95% CI, 27.1–31.4) reported perceived obstacles to treatment as reasons for not receiving treatment, and 33.8% (95% CI, 31.6–36.0) reported concerns about potential stigma associated with seeking treatment (data not shown in Tables and Figs). Observing that most of the firefighters with PTSD did not receive any treatment (Table 1 and Fig 2), we conducted an *a posteriori* analysis of the potential treatment barriers influencing absence of treatment among those with current probable PTSD, stratifying for levels of symptom severity and functional impairment. Within the subgroup of firefighters with current probable PTSD who had not received any treatment during the past month, those having greater symptom severity and functional impairment were more likely to specifically endorse concerns about potential stigma as the reason for not seeking treatment (Table 3, Tables B, C, D in S1 File).

**Table 3:** Associations of posttraumatic stress disorder (PTSD) symptom severity and perceived functional impairment with barriers to treatment in firefighters with current probable PTSD but who had not received treatment from any professional (n = 1,802)^a^.

			Concerns about potential stigma			Perceived obstacles			Joint *p* value^a^
			Adjusted OR (95% CI)		*p* value	Adjusted OR (95% CI)		*p* value	
PTSD symptom severity									
		Total PCL score, per 1-SD increase	1.16	(1.08–1.25)	<0.001	1.12	(1.05–1.21)	0.001	<0.001
		Total PCL score, per 10-point increase	1.19	(1.09–1.29)	<0.001	1.15	(1.05–1.25)	0.001	<0.001
Perceived functional impairment									
		Not at all	1.00	(referent)	-	1.00	(referent)	-	
		Somewhat	1.39	(1.04–1.86)	0.03	1.28	(0.95–1.71)	0.10	0.06
		Very	1.64	(1.20–2.25)	0.002	1.42	(1.03–1.95)	0.03	0.005
		Extremely	1.56	(1.06–2.30)	0.02	1.19	(0.80–1.75)	0.39	0.08

*Note*. PCL = Posttraumatic Stress Disorder (PTSD) Checklist; OR = odds ratio; SD = standard deviation; CI = confidence interval.

^a^ Age and rank were included as covariates, whereas years since employment was not included due to its collinearity with age (variance inflation factor = 9.1). Joint effect significance was derived from bivariate probit regression modeling [38].

## Discussion

Our findings demonstrated the 30-day rate of probable PTSD of 5.4% among public professional firefighters in South Korea. Unexpected was the high number of individuals with current probable PTSD who had not sought treatment. Perceived obstacles to treatment and concerns about potential stigma were both identified as reasons for not seeking treatment. Of note, firefighters with more severe PTSD symptoms and perceived functional impairment were more likely to specifically identify concerns about potential stigma as a barrier to treatment. This deep-seated concern of being stigmatized as ‘weak’ or ‘vulnerable’ is reported to be pervasive in firefighters [13, 42]. Paradoxically, the very qualities that make a good firefighter, e.g. perseverance, independence, courage and grit, may backfire and become one of the greatest obstacles to receiving necessary help. Therefore, special efforts are needed to address these concerns about stigma, before all else, in order to increase the awareness of mental health issues and fundamentally change the workplace narrative around PTSD.

In terms of generalizability, total population-based sampling holds significant epidemiological importance, especially for the purpose of planning and implementing effective mental health policies [43]. However, the simple extrapolations of these findings to other settings may not be appropriate. The prevalence rate of PTSD is inevitably dependent on characteristics of the study population [3, 11]. A relatively low rate (5.4%) of current probable PTSD was observed in firefighters of the current study, compared to the worldwide pooled prevalence of 10.0% in firefighters and rescue workers [3]. In the general population, the prevalence of PTSD is lower in Asian countries than other countries [44]. The 1-year prevalence of PTSD in the South Korean general population has been estimated to be 0.5% [23], which is lower than that of 3.5% in the United States [45]. Thus, the lower rate of probable PTSD observed in South Korean firefighters should be understood in the context of the correspondingly lower prevalence in the general population [23, 45]. A so-called "social desirability bias" [46–48], which is suggested to be particularly strong in East Asian cultures [23, 49–52], may also have contributed to the potential underreporting of PTSD symptoms. In addition, East Asians tend to avoid extreme expression of feelings in a self-report questionnaire than Western people due to a cultural norm [53]. However, the responses showed a high level of overall validity and the results remained unchanged even when we excluded those cases that might have been faking good, lying, or answering randomly.

Another possible factor that may have influenced the PTSD prevalence of this study may be the nature of traumatic events that firefighters had been exposed to. A previous meta-analysis suggested that firefighters and rescue workers who had been exposed to human-made disasters demonstrated a PTSD prevalence of 7.7%, whereas those who had been exposed to natural disasters showed a PTSD rate of 17.2% [3]. Although previous studies found relatively high rates of PTSD among Asian firefighters and rescue workers, these studies investigated first responders after the occurrence of large-scale natural disasters, such as earthquakes [54]. On the other hand, since our study recruited a representative nationwide population of firefighters, a substantial number of those who had not recently experienced major disasters or incidents are included, which may also account for the lower PTSD rates of this study. Morevoer, the incidence and mortality of both natural and human-made disasters that occurred in South Korea was overall lower than the worldwide trend [55].

In consistence with previous studies [5, 16], both PTSD symptom severity and perceived functional impairment due to PTSD were positively correlated with mental health service use. However, only 15.8% of firefighters with probable PTSD had received mental health treatment during the past year. This proportion is lower than that of 23% to 40% among US military personnel with depression, generalized anxiety, or PTSD [13]. Our results may reflect the lower rates of mental health service use in the South Korean general population compared to the US [23, 26]. As reasons for not receiving treatment, 29.3% of firefighters with current probable PTSD endorsed 'perceived obstacles to treatment' and 33.8% of them selected 'concerns about potential stigma'. Although similar rates of 33.1% of first responders reported stigma as a reason for not seeking treatment in a recent meta-analysis, a considerably smaller rate of 9.3% complained about practical obstacles that prevent them from receiving treatment, such as scheduling and lack of information on where to get treatment[19]. The higher rate of firefighters in our study who endorsed 'perceived obstacles to treatment' may suggest the need for an increased awareness of treatment availability. Due to the differences in definition of obstacles, sample characteristics, and social circumstances, further studies are warranted to investigate reasons behind this discrepancy. On the other hand, stigma was more closely associated with PTSD symptom severity and functional impairment than perceived obstacles in firefighters with probable PTSD but who had not received treatment. This result suggests that concerns about stigma may be a critical factor that prevents firefighters who are most in need of help from receiving adequate mental health treatment. In firefighters, fear of unintended disclosure and implicit discrimination in job assignment and promotion might have also influenced our results.

In this regard, individual resistance to treatment should be carefully considered for policies aimed at improving mental health treatment use. Regular education sessions designed to lower barriers to treatment might be helpful in changing the overall awareness and attitude toward mental health treatment [56]. Confidential outreach and employee-assistance programs directed towards de-stigmatizing mental health treatment could be of help not only for individuals with PTSD but also for those with subthreshold-level symptoms who might otherwise progress to a diagnosis of PTSD [57]. Recently developed internet and computer-based treatments have been demonstrated to be efficacious in ameliorating PTSD symptoms [58–61]. These newer approaches may provide personalized diagnosis, treatment, and facilitated patient-therapist interaction that would be more easily accessible for those who are concerned about potential stigma [60–62] or perceive insurmountable obstacles to receiving more traditional clinic or hospital-based treatment. Another policy implication would be a routine screening of firefighters with high risk for PTSD using brief assessment tools such as PCL, given that extensive evaluations may be neither feasible nor necessary for the majority of them.

Some limitations of our study should be addressesd. First, we did not account for the history of treatment among firefighters who did not endorse symptoms of PTSD. This may have led to an underestimation of PTSD, although this concern was somewhat mitigated by the low overall percentage of current treatment among this group. Second, because only those who are currently employed participated in the study there may have been a selection bias regarding those who might have resigned due to PTSD. Third, as the study participation was voluntary, it is difficult to determine whether a bias exists regarding those who chose not to participate. Nevertheless, among all currently enrolled firefighters, a high participation rate of approximately 94% was achieved in this study, alleviating the issue of nonparticipation bias [63]. Fourth, demographic variables known to affect PTSD prevalence such as education and marital status were not assessed. Nevertheless, functional impairment in family relations was included as a proxy for social support. Finally, we utilized the PCL based on the DSM-IV [29] because the PCL for DSM-5 [64] was not validated in South Korea before the study period.

The present study, to our knowledge the first country-wide, total population-based survey of South Korean firefighters, provides initial data on the rate of PTSD symptoms and barriers to treatment. We found a higher probable PTSD rate in the firefighters compared to the general population. Only a small proportion among firefighters with probable PTSD, however, had received mental health treatment during the past month due to perceived obstacles to treatment and concerns about potential stigma. Furthermore, concerns about stigma was more closely associated with both PTSD symptom severity and functional impairment. The results point to the need for new approaches promoting access to mental health treatment in professional firefighters. As different countries may have distinct cultural and social circumstances, further studies are warranted for generalization of our results.

## Supporting information

S1 File Supporting information. (DOCX)
