# Supplementary material for: Firefighters, posttraumatic stress disorder, and barriers to treatment: Results from a nationwide total population survey
Source: PLoS One. 2018 Jan 5;13(1):e0190630. doi: 10.1371/journal.pone.0190630 (PMC5755833; doi:10.1371/journal.pone.0190630)
Supplement: S1 File — (DOCX) [file pone.0190630.s001.docx]

**Supporting Information**

**Firefighters, posttraumatic stress disorder, and barriers to treatment: results from a nationwide total population survey**

**Content**

**I. Appendix A: Survey Instruments………………………………….....……...Page 2**

**II. Supplementary Analysis…………………………………........…….........…Page 9**

**III. Supplementary Tables and Figures……………………....………………..Page 10**

**IV. References for the Supporting Information…………….………...…….....Page 16**

**I. Appendix A: Survey Instruments**

| **1. Demographic and Career-Related Characteristics** | | |
| --- | --- | --- |
| **No.** | **Item** | **Response** |
| 1. | Gender | Male / Female |
| 2. | Birth year |  |
| 3. | Do you have any problems in family relationships? | Not at all / Slightly / Moderately / Extremely |
| 4. | First year of employment as a first responder |  |
| 5. | Location of workplace (affiliation) during the past 1 year |  |
| 6. | Rank | Firefighter / Senior firefighter / Fire sergeant / Fire lieutenant / Fire captain / Assistant fire chief / Fire chief / Fire marshal or higher rank |
| 7. | Major job responsibilities | Fire suppression / Emergency medical service / Rescue / Administration |

| **2. Posttraumatic Stress Disorder Checklist [1,2]^a^** | | | | | | | | | |
| --- | --- | --- | --- | --- | --- | --- | --- | --- | --- |
| "Here is a list of problems and complaints that people sometimes have in response to stressful life experiences."**^b^**  Describe the most distressing incident you have experienced on duty and answer the following questions according to that specific event.    "Please read each one carefully, and then indicate, using the numbers to the right, how much you have been bothered by that problem in the past month."**^b^** | | | | | | | | | |
| **No.** | **Item** | **Not at all** | **A little bit** | | **Moderately** | | **Quite a bit** | | **Extremely** |
| 1. | Repeated, disturbing memories, thoughts, or images of the stressful experience?**^b^** | ① | ② | | ③ | | ④ | | ⑤ |
| 2. | Repeated, disturbing dreams of the stressful experience?**^b^** | ① | ② | | ③ | | ④ | | ⑤ |
| 3. | Suddenly acting or feeling as if the stressful experience were happening again (as if you were reliving it)?**^b^** | ① | ② | | ③ | | ④ | | ⑤ |
| 4. | Feeling very upset when something reminded you of the stressful experience?**^b^** | ① | ② | | ③ | | ④ | | ⑤ |
| 5. | Having physical reactions (e.g., heart pounding, trouble breathing, or sweating) when something reminded you of the stressful experience?**^b^** | ① | ② | | ③ | | ④ | | ⑤ |
| 6. | Avoiding thinking about or talking about the stressful experience or avoiding having feelings related to it?**^b^** | ① | ② | | ③ | | ④ | | ⑤ |
| 7. | Avoiding activities or situations because they remind you of the stressful experience?**^b^** | ① | ② | | ③ | | ④ | | ⑤ |
| 8. | Trouble remembering important parts of the stressful experience?**^b^** | ① | ② | | ③ | | ④ | | ⑤ |
| 9. | Loss of interest in activities that you used to enjoy?**^b^** | ① | ② | | ③ | | ④ | | ⑤ |
| 10. | Feeling distant or cut off from other people?**^b^** | ① | ② | | ③ | | ④ | | ⑤ |
| 11. | Feeling emotionally numb or being unable to have loving feelings for those close to you?**^b^** | ① | ② | | ③ | | ④ | | ⑤ |
| 12. | Feeling as if your future will somehow be cut short?**^b^** | ① | ② | | ③ | | ④ | | ⑤ |
| 13. | Trouble falling or staying asleep?**^b^** | ① | ② | | ③ | | ④ | | ⑤ |
| 14. | Feeling irritable or having angry outbursts?**^b^** | ① | ② | | ③ | | ④ | | ⑤ |
| 15. | Having difficulty concentrating?**^b^** | ① | ② | | ③ | | ④ | | ⑤ |
| 16. | Being "super alert", watchful, or on guard?**^b^** | ① | ② | | ③ | | ④ | | ⑤ |
| 17. | Feeling jumpy or easily startled?**^b^** | ① | ② | | ③ | | ④ | | ⑤ |
|  |  | **Not difficult at all** | | **Somewhat difficult** | | **Very difficult** | | **Extremely difficult** | |
| 18. | If you checked off any problems, how difficult have these problems made it for you to do your work, take care of things at home, or get along with other people?**^c^** | ⓞ | | ① | | ② | | ③ | |
| **^a^** Prior to PCL, participants completed the Duty-related Incident Stressors Checklist which evaluates exposure to potential traumatic events that firefighters and paramedics often experience while on duty [3]. Then, they were asked to write down the most severe traumatic event they had experienced including events in that checklist or other duty-related events. Participants were asked to complete the PCL for the most severe traumatic event they had experienced on duty. **^b^** These instructions or items were adopted verbatim from the questionnaire [1,2]. **^c^** This item was adopted verbatim from the Patient Health Questionnaire-9 [4]. | | | | | | | | | |

| **3. Questions to Assess Response Validity [5]^a^** | | | |
| --- | --- | --- | --- |
| **No.** | **Item** | **Yes** | **No** |
| 1. | A nuclear war may not be such a bad idea.**^b^** |  |  |
| 2. | There are some people I don't like.**^b^** |  |  |
| 3. | Occasionally I talk about people behind their backs.**^b^** |  |  |
| 4. | I have never told a lie.**^b^** |  |  |
| 5. | Sometimes I get upset.**^b^** |  |  |
| 6. | I have lied a lot on this questionnaire.**^b^** |  |  |
| **^a^** These questions were adapted from the Personality Diagnostic Questionnaire-IV [5]. If the participants answered "yes" to questions 1 or 6 then the possibility of them lying or answering randomly on the questionnaire was considered. Participants received 1 point if they answered "yes" on question 4 and "no" on questions 2, 3 and 5. If the sum of the four questions was greater than 3 then the possibility of them answering the questionnaire in a way that will be viewed favorably by others was considered. These items were interspersed with other items throughout the questionnaire. **^b^** These instructions or items were adopted verbatim from the questionnaire [5]. | | | |

| **4. Utilization of Mental Health Treatment Services [6,7]** | | | | |
| --- | --- | --- | --- | --- |
| **No.** | **Item** | **Yes** | | **No** |
| 1. | Have you received any professional treatment (from a psychiatrist, a general medical doctor, a religious mentor, a counselor^a^, etc.) such as medical treatment or psychological counseling for mental health problems (depressive mood, stress, anxiety, insomnia, etc.) in the past year? |  | |  |
|  | 1-1. If you replied with a "yes" to Item 1, have you received treatment from a psychiatrist? |  | |  |
|  | 1-2. If you replied with a "no" to Item 1-1, from whom have you received help? | Free response | | |
| 2. | Have you received any professional treatment (from a psychiatrist, a general medical doctor, a religious mentor, a counselor^a^, etc.) in the past month? |  |  | |
|  | 2-1. If you replied with a "yes" to Item 2, have you received treatment from a psychiatrist? |  |  | |
|  | 2-2. If you replied with a "no" to Item 2-1, from whom have you received treatment? | Free response | | |
| ^a^ Psychologists and psychotherapists were included in this term. | | | | |

| **5. Barriers to Treatment [6]** | | |
| --- | --- | --- |
| Why did you choose not to get treatment, or why were you unable to get treatment, on issues related to mental health (depressive mood, stress, anxiety, insomnia, etc.)?  Please check all the items that apply (multiple choice). | | |
| **No.** | **Item** | **Yes** |
| 1. | I don’t think I have a mental health problem. |  |
| 2. | I have some difficulties associated with a mental health problem, but I never felt the need for any professional help. |  |
| 3. | I didn't know that I could receive help for these matters. |  |
| 4. | I don’t know where to get help.**^a^** |  |
| 5. | I don't have enough time to spend on treatment. |  |
| 6. | Mental health care costs too much money.**^a^** |  |
| 7. | It would harm my career.**^a^** |  |
| 8. | I would be seen as weak.**^a^** |  |
| **^a^** These items were adopted verbatim from Hoge et al [6]. | | |

**II. Supplementary Analysis**

**Supplementary Method A**

In addition to a total PTSD Checklist (PCL) score of 45 [1], we also estimated the 30-day prevalence of probable PTSD using an algorithm-derived PTSD diagnosis method as a sensitivity analysis. Among the 17 items of the PCL, the first 5 items correspond to the Diagnostic and Statistical Manual of Mental Disorders-IV (DSM-IV) [8] diagnosis criteria of “B” (re-experiencing), the next 7 to criteria “C” (avoidance/numbing), and the last 5 to criteria “D” (hyperarousal). While the cutoff method calculates a total score, the algorithm-derived PTSD diagnosis requires a rating of 3 or more (in a 5 point Likert scale) for at least one "re-experiencing” symptom, three “avoidance/numbing” symptoms, and two “hyperarousal” symptoms.

**Supplementary Results A**

In the main analysis using the cutoff score of 45, the 30-day prevalence of probable PTSD was 5.4% (*n*=1,995; 95% confidence interval [CI], 5.1–5.6) in Model 1, 6.0% (*n*=1,979; 95% CI, 5.8–6.3) in Model 2, and 5.5% (*n*= 1,932; 95% CI, 5.3–5.8) in Model 3. When using the DSM-IV algorithm-derived PTSD diagnosis for PCL, the prevalence of probable PTSD was 6.4% (*n* = 2,086; 95% confidence interval [CI], 6.1–6.6) in Model 2.

**III. Supplementary Tables and Figures**

**Table A.** **Characteristics of firefighters with or without current probable posttraumatic stress disorder, in the sample where missing values were pairwise deleted^a^**

| **Characteristic** | | | **PTSD group**  (*n*=1,979) | | | | **Non-PTSD group**  (*n*=30,962) | | | | **Test statistics^b^** |
| --- | --- | --- | --- | --- | --- | --- | --- | --- | --- | --- | --- |
| Demographic | | |  |  | |  |  |  | |  |  |
|  | Age, years | | 42.6 | ± | | 8.0^c^ | 41.2 | ± | | 8.5^d^ | *p* < .001, d = 0.17, CI = 0.13-0.22 |
|  | Male, *N* (%) | | 1,794 | | (94.0)^e^ | | 28,282 | | (94.6)^f^ | | *p* = .22, *OR* = 1.13, CI = 0.93-1.37, |
| Career-related | | |  | |  | |  | |  | |  |
|  | Higher rank, *N* (%)^g^ | | 1,263 | | (64.1)^h^ | | 17,522 | | (56.8)^i^ | | *p* < .001, *OR* = 1.36, CI = 1.23-1.49 |
|  | Working at cities, *N* (%)^j^ | | 1,224 | | (61.9) | | 19,303 | | (62.3) | | *p* = .66, *OR* = 0.98, CI = 0.89-1.08 |
|  | Years since employment, years | | 16.0 | ± | | 8.3^k^ | 14.5 | ± | | 8.7^l^ | *p* < .001, d = 0.18, CI = 0.13-0.22 |
| Clinical | | |  |  | |  |  |  | |  |  |
|  | Total PCL score | | 55.1 | ± | | 9.0^m^ | 21.1 | ± | | 6.5^n^ | *p* < .001, d = 5.12, CI = 5.06-5.19 |
|  | Received treatment during the past year | |  | |  | |  | |  | |  |
|  |  | From any professional, *N* (%) | 300 | | (15.3)^k^ | | 806 | | (2.6)^o^ | | *p* < .001, *OR* = 6.71, CI = 5.83-7.73 |
|  |  | From a psychiatrist, *N* (%) | 183 | | (9.6)^p^ | | 436 | | (1.4)^q^ | | *p* < .001, *OR* = 7.33, CI = 6.13-8.77 |
|  | Received treatment during the past month | |  | |  | |  | |  | |  |
|  |  | From any professional, *N* (%) | 186 | | (9.5)^k^ | | 329 | | (1.1)^r^ | | *p* < .001, *OR* = 9.70, CI = 8.05-11.68 |
|  |  | From a psychiatrist, *N* (%) | 124 | | (6.4)^s^ | | 198 | | (0.6)^t^ | | *p* < .001, *OR* = 10.59, CI = 8.42-13.32 |
|  | Functional impairment in family relations | |  | |  | |  | |  | | *p* < .001, *OR* = 9.66, CI = 8.82-10.59 |
|  |  | Not at all | 694 | | (35.4)^u^ | | 25,258 | | (82.2)^v^ | |  |
|  |  | Slightly | 761 | | (38.8)^u^ | | 4,684 | | (15.3)^v^ | |  |
|  |  | Moderately | 364 | | (18.6)^u^ | | 543 | | (1.8)^v^ | |  |
|  |  | Extremely | 141 | | (7.2)^u^ | | 239 | | (0.8)^v^ | |  |

*Note.* OR = odds ratio; CI = 95% confidence interval; PCL = Posttraumatic Stress Disorder (PTSD) Checklist [2].

^a^ Diagnosis of current probable PTSD is based on the PCL (total score ≥ 45). ^b^ Independent t-tests or chi-square tests were used. Effect size was estimated using Cohen's d or odds ratio. ^c^ *n* = 1,972. ^d^ *n* = 30,906. ^e^ *n* = 1,909. ^f^ *n* = 29,888. ^g^ Fire captain or higher. ^h^ *n* = 1,971. ^i^ *n* = 30,832. ^j^ Locations with a population greater than 380,000 were defined as cities [9,10]. ^k^ *n* = 1,961. ^l^ *n* = 30,808. ^m^ *n* = 1,923. ^n^ *n* = 30,622. ^o^ *n* = 30,761. ^p^ *n* = 1,911. ^q^ *n* = 30,624. ^r^ *n* = 30,778. ^s^ *n* = 1,929. ^t^ *n* = 30,725. ^u^ *n* = 1,960. ^v^ *n* = 30,724.

**Table B. Associations of posttraumatic stress disorder (PTSD) symptom severity and perceived functional impairment with barriers to treatment in firefighters with current probable PTSD who had not received treatment in the sample where missing values were pairwise deleted (n=1,608)^a^**

|  | | | **Concerns about potential stigma** | | |  | **Perceived obstacles** | | | **Joint *p* value**^b^ |
| --- | --- | --- | --- | --- | --- | --- | --- | --- | --- | --- |
|  |  |  | **Adjusted OR (95% CI)** | | ***p* value** |  | **Adjusted OR (95% CI)** | | ***p* value** |  |
| PTSD symptom severity | | |  |  |  |  |  |  |  |  |
|  | | Total PCL score, per 1-SD increase | 1.18 | (1.09 – 1.27) | < .001 |  | 1.14 | (1.06 – 1.23) | .001 | < .001 |
|  | | Total PCL score, per 10-point increase | 1.21 | (1.11 – 1.32) | < .001 |  | 1.17 | (1.07 – 1.27) | .001 | < .001 |
| Perceived functional impairment | | |  |  |  |  |  |  |  |  |
|  | Not at all | | 1.00 | (referent) | - |  | 1.00 | (referent) | - | - |
|  | Somewhat | | 1.38 | (1.03 – 1.85) | .03 |  | 1.26 | (0.94 – 1.69) | .12 | .07 |
|  | Very | | 1.64 | (1.19 – 2.27) | .003 |  | 1.41 | (1.02 – 1.95) | .04 | .01 |
|  | Extremely | | 1.69 | (1.14 – 2.49) | .01 |  | 1.13 | (0.76 – 1.68) | .56 | .03 |

*Note.* PCL = Posttraumatic Stress Disorder (PTSD) Checklist [2]; OR = odds ratio; SD = standard deviation; CI = confidence interval.

^a^ Results from sensitivity analyses using pairwise deletion methods for missing data, in the subsample of firefighters who had not received treatment from any professional. ^b^ Age and rank were included as covariates, whereas years since employment was not included due to its collinearity with age (variance inflation factor = 9.1). Joint effect significance was derived from bivariate probit regression modeling.

**Table C. Associations of posttraumatic stress disorder (PTSD) symptom severity and perceived functional impairment with barriers to treatment in firefighters with current probable PTSD who had not received treatment, in the sample where cases were excluded according to response validity criteria (n=1,761)^a^**

|  | | | **Concerns about potential stigma** | | |  | **Perceived obstacles** | | | **Joint *p* value**^b^ |
| --- | --- | --- | --- | --- | --- | --- | --- | --- | --- | --- |
|  |  |  | **Adjusted OR (95% CI)** | | ***p* value** |  | **Adjusted OR (95% CI)** | | ***p* value** |  |
| PTSD symptom severity | | |  |  |  |  |  |  |  |  |
|  | | Total PCL score, per 1-SD increase | 1.16 | (1.08 – 1.24) | < .001 |  | 1.12 | (1.05 – 1.21) | .002 | < .001 |
|  | | Total PCL score, per 10-point increase | 1.19 | (1.09 – 1.29) | < .001 |  | 1.15 | (1.05 – 1.25) | .002 | < .001 |
| Perceived functional impairment | | |  |  |  |  |  |  |  |  |
|  | Not at all | | 1.00 | (referent) | - |  | 1.00 | (referent) | - | - |
|  | Somewhat | | 1.39 | (1.04 – 1.85) | .03 |  | 1.27 | (0.95 – 1.69) | .11 | .06 |
|  | Very | | 1.65 | (1.21 – 2.27) | .002 |  | 1.40 | (1.02 – 1.93) | .04 | .005 |
|  | Extremely | | 1.59 | (1.08 – 2.36) | .02 |  | 1.19 | (0.81 – 1.75) | .38 | .07 |

*Note.* PCL = Posttraumatic Stress Disorder (PTSD) Checklist [2]; OR = odds ratio; SD = standard deviation; CI = confidence interval.

^a^ Results from sensitivity analyses using data sets excluding individuals who could be faking good, answering randomly, or lying, using questions adapted from the Personality Diagnostic Questionnaire-IV. ^b^ Age and rank were included as covariates, whereas years since employment was not included due to its collinearity with age (variance inflation factor = 9.1). Joint effect significance was derived from bivariate probit regression modeling.

**Table D. Associations of posttraumatic stress disorder (PTSD) symptom severity and perceived functional impairment with barriers to treatment in firefighters with current probable PTSD who had not received treatment from a psychiatrist (n=1,840)**

|  | | | **Concerns about potential stigma** | | |  | **Perceived obstacles** | | | **Joint *p* value**^a^ |
| --- | --- | --- | --- | --- | --- | --- | --- | --- | --- | --- |
|  |  |  | **Adjusted OR (95% CI)** | | ***p* value** |  | **Adjusted OR (95% CI)** | | ***p* value** |  |
| PTSD symptom severity | | |  |  |  |  |  |  |  |  |
|  | | Total PCL score, per 1-SD increase | 1.17 | (1.09 – 1.25) | < .001 |  | 1.12 | (1.04 – 1.20) | .003 | < .001 |
|  | | Total PCL score, per 10-point increase | 1.20 | (1.10 – 1.30) | < .001 |  | 1.14 | (1.05 – 1.24) | .003 | < .001 |
| Perceived functional impairment | | |  |  |  |  |  |  |  |  |
|  | Not at all | | 1.00 | (referent) | - |  | 1.00 | (referent) | - | - |
|  | Somewhat | | 1.46 | (1.09 – 1.95) | .01 |  | 1.34 | (1.00 – 1.80) | .05 | .02 |
|  | Very | | 1.72 | (1.26 – 2.35) | .001 |  | 1.49 | (1.08 – 2.05) | .02 | .002 |
|  | Extremely | | 1.63 | (1.12 – 2.38) | .01 |  | 1.31 | (0.89 – 1.93) | .17 | .04 |

*Note.* PCL = Posttraumatic Stress Disorder (PTSD) Checklist [2]; OR = odds ratio; SD = standard deviation; CI = confidence interval.

^a^ Age and rank were included as covariates, whereas years since employment was not included due to its collinearity with age (variance inflation factor = 9.1). Joint effect significance was derived from bivariate probit regression modeling.


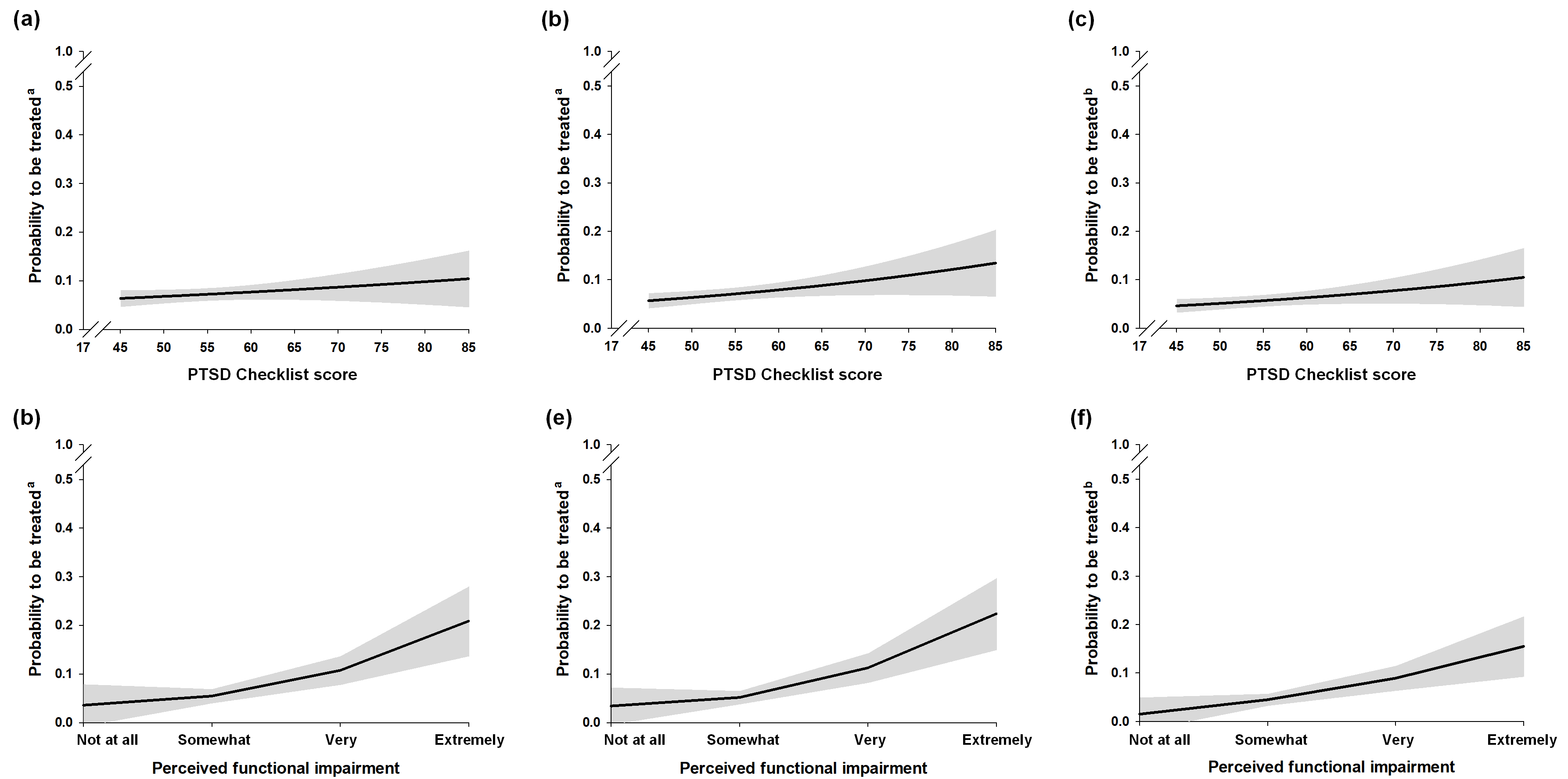


**Fig A. Mental health treatment use during the past month according to posttraumatic stress disorder (PTSD) symptom severity (a-c) and degree of perceived functional impairment (d-f).** (a) and (d) are from pairwise deleted datasets (Model 2 of Figure 1), while (b) and (e) are from datasets excluding cases according to response validity criteria (Model 3 of Figure 1). (c) and (f) are from multivariate imputed datasets with the treatment use from a psychiatrist as the dependent variable. ^a^ Treatment from any professional. ^b^ Treatment from a psychiatrist.

**IV. References for the Supporting Information**

1. Park S, Jeong HS, Im JJ, Jeon Y, Ma J, et al. (2016) Reliability and validity of the Korean version of the PTSD checklist in firefighters and rescue workers. Korean J Biol Psychiatry 23: 29-36.

2. Weathers FW, Litz BT, Herman DS, Huska JA, Keane TM (1993) The PTSD Checklist (PCL): reliability, validity, and diagnostic utility. Annual Meeting of International Society for Traumatic Stress Studies. San Antonio: International Society for Traumatic Stress Studies.

3. Beaton R, Murphy S, Johnson C, Pike K, Corneil W (1998) Exposure to duty-related incident stressors in urban firefighters and paramedics. J Trauma Stress 11: 821-828.

4. Kroenke K, Spitzer RL, Williams JB (2001) The PHQ-9: validity of a brief depression severity measure. J Gen Intern Med 16: 606-613.

5. Hyler S (1994) Personality Diagnostic Questionnaire-4. New York: New York State Psychiatric Institute.

6. Hoge CW, Castro CA, Messer SC, McGurk D, Cotting DI, et al. (2004) Combat duty in Iraq and Afghanistan, mental health problems, and barriers to care. N Engl J Med 351: 13-22.

7. Kim PY, Thomas JL, Wilk JE, Castro CA, Hoge CW (2010) Stigma, barriers to care, and use of mental health services among active duty and National Guard soldiers after combat. Psychiatr Serv 61: 582-588.

8. American Psychiatric Association (2000) Diagnostic and Statistical Manual of Mental Disorders. 4th ed. Text Revision. Washington, DC: American Psychiatric Association.

9. Central Intelligence Agency (2015) The World Factbook.

10. Mortensen PB, Pedersen CB, Westergaard T, Wohlfahrt J, Ewald H, et al. (1999) Effects of family history and place and season of birth on the risk of schizophrenia. N Engl J Med 340: 603-608.
